# Newborn screening for severe combined immunodeficiency in Malaysia: current status, challenges and progress

**DOI:** 10.3389/fimmu.2024.1456769

**Published:** 2024-10-02

**Authors:** Wai Leng Chang, Lokman Mohd Noh, Amir Hamzah Abdul Latiff, Kent Chee Keen Woo, Intan Hakimah Ismail, Intan Juliana Abd Hamid, Sangeetha Siniah, Mohd Azri Zainal Abidin, Marina Sham, Adiratna Mat Ripen, Mohd Farid Baharin, Asrul Abdul Wahab, Zarina Thasneem Zainudeen, Ilie Fadzilah Hashim, Yee Ming Wong, Mohamad Qazreen Ahmad Shawaludin, Adli Ali

**Affiliations:** ^1^ Department of Pediatric, Faculty of Medicine, Universiti Kebangsaan Malaysia, Kuala Lumpur, Malaysia; ^2^ Research Center, Hospital Tunku Ampuan Besar Tuanku Aishah Rohani, Universiti Kebangsaan Malaysia (UKM) Specialist Children’s Hospital, Kuala Lumpur, Malaysia; ^3^ Malaysia Patient Organization for Primary Immunodeficiencies (MYPOPI), Shah Alam, Selangor, Malaysia; ^4^ Allergy and Immunology Centre, Pantai Hospital Kuala Lumpur, Kuala Lumpur, Malaysia; ^5^ Sunway Centre for Planetary Health, Sunway University, Petaling Jaya, Malaysia; ^6^ Allergy and Immunology, Gleneagles Hospital, Kuala Lumpur, Malaysia; ^7^ Clinical Immunology Unit, Department of Paediatrics, Faculty of Medicine and Health Sciences, Universiti Putra Malaysia, Serdang, Malaysia; ^8^ Primary Immunodeficiency Diseases Group, Department of Clinical Medicine, Institut Perubatan & Pergigian Termaju, Universiti Sains Malaysia, Kepala Batas, Pulau Pinang, Malaysia; ^9^ Pediatric Infectious Disease and Immunology Unit, Department of Pediatric, Hospital Tunku Azizah, Kuala Lumpur, Malaysia; ^10^ Cancer Research Centre, Institute for Medical Research (IMR), National Institutes of Health (NIH), Shah Alam, Malaysia; ^11^ Allergy and Immunology Research Centre, Institute for Medical Research (IMR), National Institutes of Health (NIH), Shah Alam, Malaysia; ^12^ Department of Medical Microbiology and Immunology, Faculty of Medicine, Universiti Kebangsaan Malaysia, Kuala Lumpur, Malaysia; ^13^ Department of Pediatrics, Columbia Asia Hospital Bukit Jalil, Kuala Lumpur, Malaysia; ^14^ Department of Pediatrics, Hospital Enche Besar Hajjah Khalsom, Kluang, Johor, Malaysia; ^15^ Institute of IR4.0, Universiti Kebangsaan Malaysia, Bangi, Malaysia; ^16^ Infection and Immunology Health and Advanced Medicine Cluster, Universiti Kebangsaan Malaysia, Kuala Lumpur, Malaysia

**Keywords:** neonatal, immunodeficiency, implementation, Malaysia, strategy, advances, barriers

## Abstract

**Introduction:**

Early diagnosis of Severe Combined Immunodeficiency (SCID) increases survival outcomes and quality of life while significantly minimizing healthcare burden and costs. Despite growing evidence supporting the benefits and cost-effectiveness of SCID detection through newborn screening (NBS), it has yet to be implemented in Malaysia. This study aims to explore experts’ opinions on the current status, challenges, and crucial strategies needed for the successful implementation of SCID NBS.

**Methodology:**

A guided, structured interview was employed to explore opinions on the current status, barriers, and strategies for implementing SCID NBS in Malaysia. All 13 invited experts participated in this study, indicating complete participation from the entire Malaysian immunology fraternity (consisting of eight clinical immunologists and five immunopathologists).

**Key findings:**

Several initiatives are ongoing to establish SCID NBS in Malaysia. Hindrances such as low immunologist-to-patient ratio, unequal placements of immunologists throughout Malaysia, society’s low disease awareness, national health prioritization, lack of stakeholder engagement, and inadequacy of local study/data were highlighted. Pilot research on SCID NBS, advocacy workshops, and promotion materials are among the ongoing activities outlined in the blueprint, paving the way for this nationwide NBS program to be achievable in the near future.

**Conclusion:**

This article provides recommendations to policymakers in mandating SCID NBS. Strategies by key stakeholders are underway, particularly in advocacy programs and efforts to increase awareness among clinicians and the public.

## 1 Introduction

Primary Immunodeficiency Diseases (PID) or Inborn Errors of Immunity (IEI) are defined as a heterogeneous group of hereditary disorders caused by genetic mutations, contributing to defective development and malfunction of the immune system (1, 25). Severe Combined Immunodeficiency (SCID) is the most devastating form of PID, as SCID patients exhibit defects in the formation or differentiation of T lymphocytes with variable effects on B and NK lymphocytes, exacerbating the associated problems (2). SCID patients are asymptomatic at birth (3). They may present with severe, recurrent infections caused by various pathogens, manifesting as faltering growth, chronic diarrhea, recurrent pneumonia, and, in some cases, infections after receiving live vaccines in the first few months of life (4–6). The incidence of SCID was originally estimated at 1 in 100,000 neonates. Following the implementation of SCID newborn screening (NBS) in the United States, the increased incidence was estimated at 1 in 58,000 (7).

Early detection of SCID through SCID NBS is essential as higher survival rates are seen in children who have received HSCT infection-free (8, 9). SCID is typically fatal within the first year of life unless patients receive definitive treatment, such as a hematopoietic stem cell transplant (HSCT), and in specific genetic variants of SCID, enzyme replacement therapy, or gene therapy, with better survival outcomes are associated with early curative treatment before the age of 3.5 months old (10). NBS for SCID is conducted by quantifying T-cell receptor excision circles (TRECs) in dried blood spots using quantitative polymerase chain reaction (qPCR). TRECs are excised DNA fragments formed during T lymphocyte differentiation, directly proportional to the amount of gene rearrangement or T cell receptor (TCR) rearrangement in the thymus (11). As a low level of TRECs is seen in SCID and other non-SCID T-cell lymphopenia, further testing and evaluation with an immunologist are warranted following abnormal NBS results (12). TREC is a good screening tool as it has high sensitivity and specificity. Moreover, curative treatment can be instituted early for SCID patients, significantly reducing mortality (13).

Although it is well-known that NBS using TREC and/or kappa-deleting recombination excision circle (KREC) is an effective public health measure to effectively screen for SCID and other possible IEIs, Malaysia, on its way to becoming a developed nation, has yet to embark on such NBS program. Globally, 42 countries already have SCID NBS initiatives. Among them, seven are developing countries (14). SCID NBS, like other NBS programs, should be integrated, even more so following the recent adoption of the NBS resolution in the 77^th^ World Health Assembly (WHA). The WHA is the decision-making body of the World Health Organization which determines organization policies, appoints the Director-General, supervises financial policies, and reviews and approves the proposed program budget. Thus, this study explores the current status of the integration of SCID NBS in Malaysia, the barriers, and the strategies that should be employed to ensure its successful implementation.

## 2 Materials and methods

A guided, structured interview was employed to explore the opinions of Malaysian-based experts on the implementation of SCID NBS in Malaysia. Study invitations were sent to all 13 Malaysian immunologists and immunopathologists, except for one immunologist, the principal investigator, who was excluded from participating. All the invited experts participated in this study. The questions were derived from the literature review of research articles and further refined through focused-group discussions attended by clinical immunologists, immunopathologists, clinical geneticists, eight patient support group representatives, and delegates from the Ministry of Health (MOH). Specifically, the experts were asked about the current status, challenges, and strategies that should be employed in implementing SCID NBS.

Qualitative analysis was employed, and emerging themes were coded and analyzed. The questions asked during the interview were open-ended, hence the experts could freely provide opinions on the challenges and strategies in implementing SCID NBS. The duration of interview ranged between 30-40 minutes for each respondent.

This study was approved by the Research Ethics Committee, The National University of Malaysia (UKM PPI/111/8/JEP-2023-888), and complied with the Declaration of Helsinki and Malaysian Good Clinical Practice Guideline.

## 3 Key findings

### 3.1 Current status of NBS for SCID in Malaysia

Newborns in Malaysia are readily screened for glucose-6-phosphate dehydrogenase (G6PD) deficiency and congenital hypothyroidism using cord blood in the nationwide NBS program. The first documented and published SCID case in Malaysia was in 1993 (15). Previously, due to inadequate clinical immunologists, a lack of awareness of SCID, and insufficient diagnostic facilities, SCID cases were underdiagnosed. With increased awareness among healthcare professionals and an increasing number of clinical immunologists, SCID cases have been diagnosed consistently annually, with the highest number of SCID reported in 2013 being seven cases. The most recent data indicated five reported and confirmed cases of SCID in Malaysia in 2023. Based on an estimated 423,124 live births in Malaysia in 2022 (16) and the probable documented incidence rate of 1 in 58,000 infants for SCID (7), approximately eight new cases of SCID are expected annually in Malaysia. Hence, this suggests that at least three SCID cases were missed. This aligns with the input from one of the experts who shared two reported cases of suspected SCID referred to their center in the same year. Unfortunately, these patients passed away before a SCID diagnosis could be ascertained.

In Malaysia, the first haploidentical HSCT was successfully conducted in 2014. There is a cord blood bank that can be utilized for HSCT. However, allogenic haploidentical HSCT for SCID is the current local practice. Matched unrelated donors (MUD) HSCT have yet to become the norm. This is because there is a low probability (2-5%) of obtaining a MUD from the Malaysian national donor registry, attributed to a relatively small registry pool and ethnic heterogeneity. Referrals for HSCT with MUD are usually made to Singapore, Thailand and India. HSCT for IEI is associated with a 90% overall survival rate in Malaysia (17, 18). Meanwhile, adenosine deaminase (ADA) enzyme treatment is currently unavailable.

The experts generally expressed varied opinions on the feasibility of launching a national SCID NBS program. 53.8% experts believe it is achievable, provided certain prerequisites are met. These include (i) proper planning, (ii) the availability and adequacy of infrastructure and manpower, (iii) proof of cost-effectiveness, and (iv) the extraction of insightful data from pilot studies of SCID NBS in Malaysia.

Five experts (38.5%) express optimism that it can be implemented as Malaysia has successful NBS programs on G6PD deficiency and congenital hypothyroidism. This success suggests that incorporating SCID into the existing screening panel could be easily achieved. There is strong advocacy from NGOs such as the Malaysia Patient Organization for Primary Immunodeficiencies (MyPOPI) and the International Patient Organization for Primary Immunodeficiencies (IPOPI), as well as relevant authorities in the MOH, all aligned with its implementation.

However, one immunologist is less optimistic, suggesting that the implementation of SCID NBS is unlikely. This perspective is grounded in classifying SCID as a rare disease that currently lacks recognition in Malaysia.

### 3.2 Challenges toward implementing national SCID NBS

#### 3.2.1 Manpower and expertise shortage and uncertain commitment among healthcare workers

**Figure 1:**
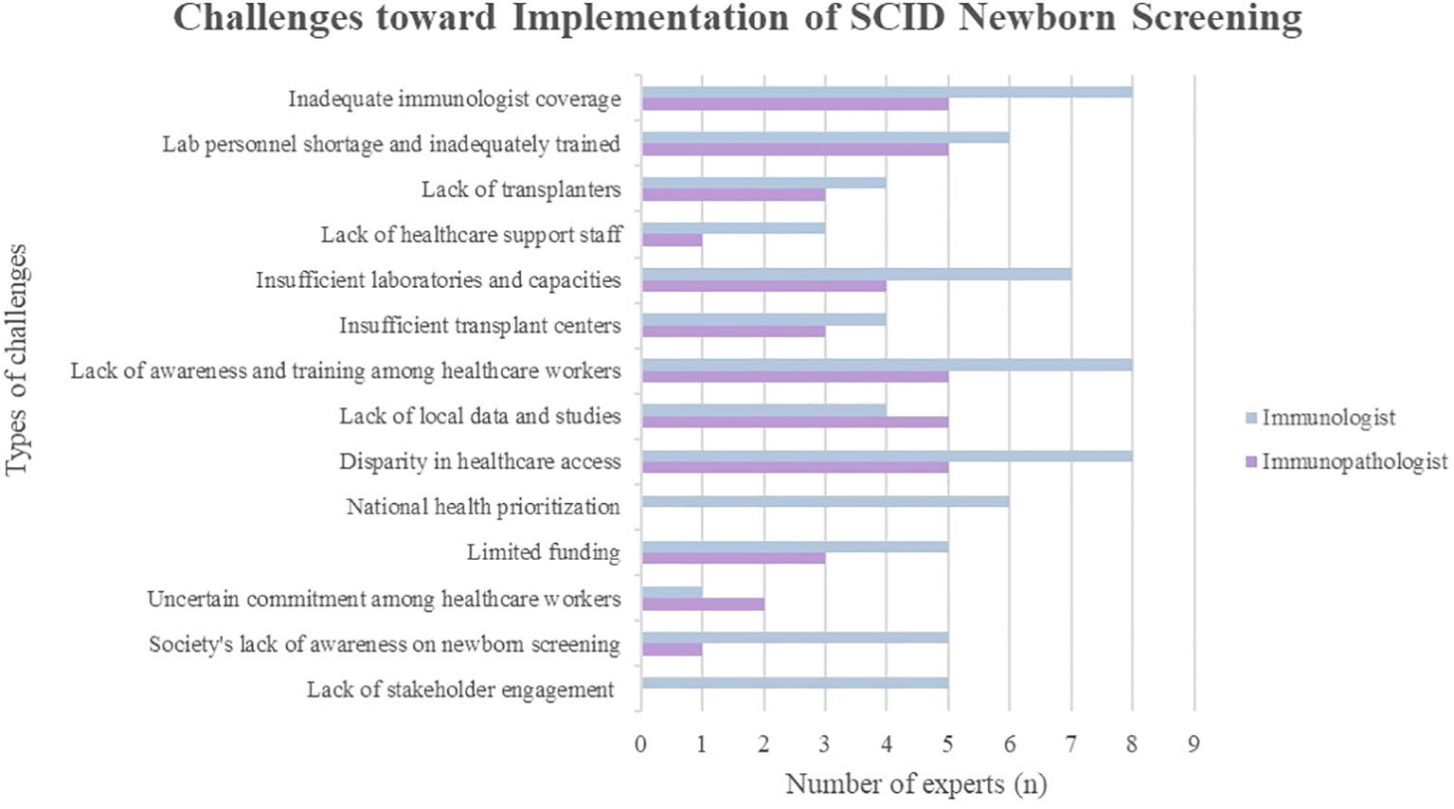
Challenges toward implementation of SCID newborn screening in Malaysia.

All experts (100%) acknowledged insufficient immunologists as one of the main challenges (**Figure 1**). Malaysia’s population is estimated at 33.4 million in 2023 which makes the ratio of one clinical immunologist to 3.71 million of the Malaysian population. The recommended ratio is two immunologists per one million population (19). There are only nine immunologists in Malaysia, with eight specializing in pediatric immunology and one in adult immunology. The adult immunologist serves in the private sector, leading to the absence of adult immunology services in government hospitals. Most pediatric immunologists are based in university hospitals, primarily in Selangor or Kuala Lumpur in Peninsular Malaysia. Only one pediatric immunologist serves outside central Peninsular Malaysia, at an academic hospital covering the northern and east coast regions. There are no permanent immunologists based in other states in Peninsular Malaysia, and services are yet to commence in East Malaysia. Consequently, many PID patients are managed by other pediatric subspecialties (20), posing a risk of PID patients being misdiagnosed or undiagnosed, leading to serious health sequelae and mortality (21).

There is inadequate laboratory personnel and they are insufficiently trained to conduct and interpret SCID NBS tests. Furthermore, the inadequacy of transplanters was underscored. Presently, only three pediatric HSCT specialists serve in Malaysia. They are responsible for the HSCT for various conditions, lacking specialization in HSCT for SCID. Given the anticipated patient increase and the stagnant number of pediatric transplant specialists, there is concern about longer waiting times for HSCT if SCID NBS is implemented. Additionally, there is potential inadequacy of nurses and genetic counselors in managing SCID patients.

The uncertain commitment among healthcare workers is evident. With a lack of priority given to the management of immunological diseases, particularly in the field of primary immunodeficiency, there is an additional burden on healthcare workers beyond their original job scope. This includes specimen collection, tracking and contacting patients for retesting or confirmatory tests.

#### 3.2.2 Inadequate infrastructure

Most experts expressed concerns about insufficient laboratories and capacities, including space and specific instruments, to cater to the increased testing demands expected with the implementation of SCID NBS. These existing laboratories are yet to be accredited; moreover, accreditation may be challenging due to the long process and high costs. The Standards Malaysia accreditation system requires medical laboratories to have International Organization for Standardization (ISO)-accredited standards. Besides, despite the availability of genetic testing technology and laboratories locally, the higher operational costs and longer turnover time lead to outsourcing genetics analyses to foreign laboratories. As earlier highlighted, referral centers with clinical immunology expertise are concentrated in Kuala Lumpur and Selangor. Furthermore, the need for more transplant centers is evident. Currently, HSCT for SCID patients is exclusively performed in two hospitals in Kuala Lumpur, Malaysia’s capital city.

#### 3.2.3 Lack of awareness and inadequately trained healthcare workers

Another significant hindrance is the lack of awareness regarding PID and the importance of SCID NBS among Malaysian healthcare workers and the general public. The public is deemed unaware of the availability of NBS for conditions beyond those currently in place. During an expert meeting involving other pediatric subspecialties and experts, non-immunologists’ input on NBS for IEI was limited due to a lack of awareness of the prevalence, diagnosis, and management of IEI.

Additionally, a majority of healthcare professionals lack sufficient training to manage PID patients, presenting a dual challenge. Newborns in Malaysia are screened for G6PD deficiency and congenital hypothyroidism, both using cord blood. Hence, most healthcare workers might be inadequately trained to perform heel pricking and collect specimens.

#### 3.2.4 Absence of national registry and lack of local studies

Some experts believe that the lack of a national registry hampers the commencement of SCID NBS as this data is useful in providing recommendations to policymakers. The lack of research studies is attributed to insufficient clinicians specializing in immunology, particularly in translational efforts. Notably, countries with dense populations, such as China and India, have reported low estimated diagnosis rates compared to their population sizes due to the lack of national registries (22).

Nevertheless, one immunologist opined that the scarcity of local studies does not preclude the necessity of SCID NBS. Instead, its implementation may contribute to a more accurate determination of SCID prevalence and incidence rates. Data integration is currently underway and the establishment of a national registry is in the pipeline.

#### 3.2.5 Disparity in healthcare access

The disparity in healthcare access was a concern raised by all the experts (100%), driven by geographical barriers, financial considerations, and an unequal distribution of healthcare manpower and resources. Residents in rural areas and East Malaysia encounter difficulties in accessing healthcare due to poor road conditions and infrastructure. Established tertiary hospitals are more comprehensively equipped with specialists and laboratory facilities than smaller government hospitals in the district’s region. This imbalance is attributed to a continual migration of experienced doctors from the government sector to private practices, reducing the number of clinicians in the public sector. In addition, these specialized services are primarily concentrated in urban areas. Clinical immunology had inadequate support compared to other medical disciplines, leading to misdiagnosis and unsubsidized treatments. Access to private healthcare institutions is constrained by one’s financial status and insurance coverage availability. Families shoulder the full charges for PID confirmatory tests in private hospitals without insurance coverage, as PID is considered a congenital disease and, thus, excluded from insurance coverage (20). Patients with lower socioeconomic status face the risk of poorer health outcomes as they are deprived of options in obtaining healthcare services (23). The government’s lower spending on health (calculated as a percentage of Gross Domestic Product) than proposed also contributes to the inequality in healthcare access.

#### 3.2.6 National health prioritization, limited funding, and lack of stakeholder engagement

The presence of other competing health priorities hinders the integration of SCID NBS, according to six immunologists (75%). The government’s lack of recognition of SCID mirrors the case of other rare diseases. Despite the recent establishment of a national rare disease working committee under the MOH office, the entity is still at its early formation stage with very limited funding. Additionally, there is limited government funding to conduct NBS and diagnostic tests, hire healthcare staff, and provide training and infrastructure.

As the field of PID currently lacks prioritization, there is inadequate discussion, collaboration, and planning involving healthcare professionals, patient support groups, policymakers, and the public for the implementation of SCID NBS.

### 3.3 Employing strategies to embark on SCID NBS

**Figure 2:**
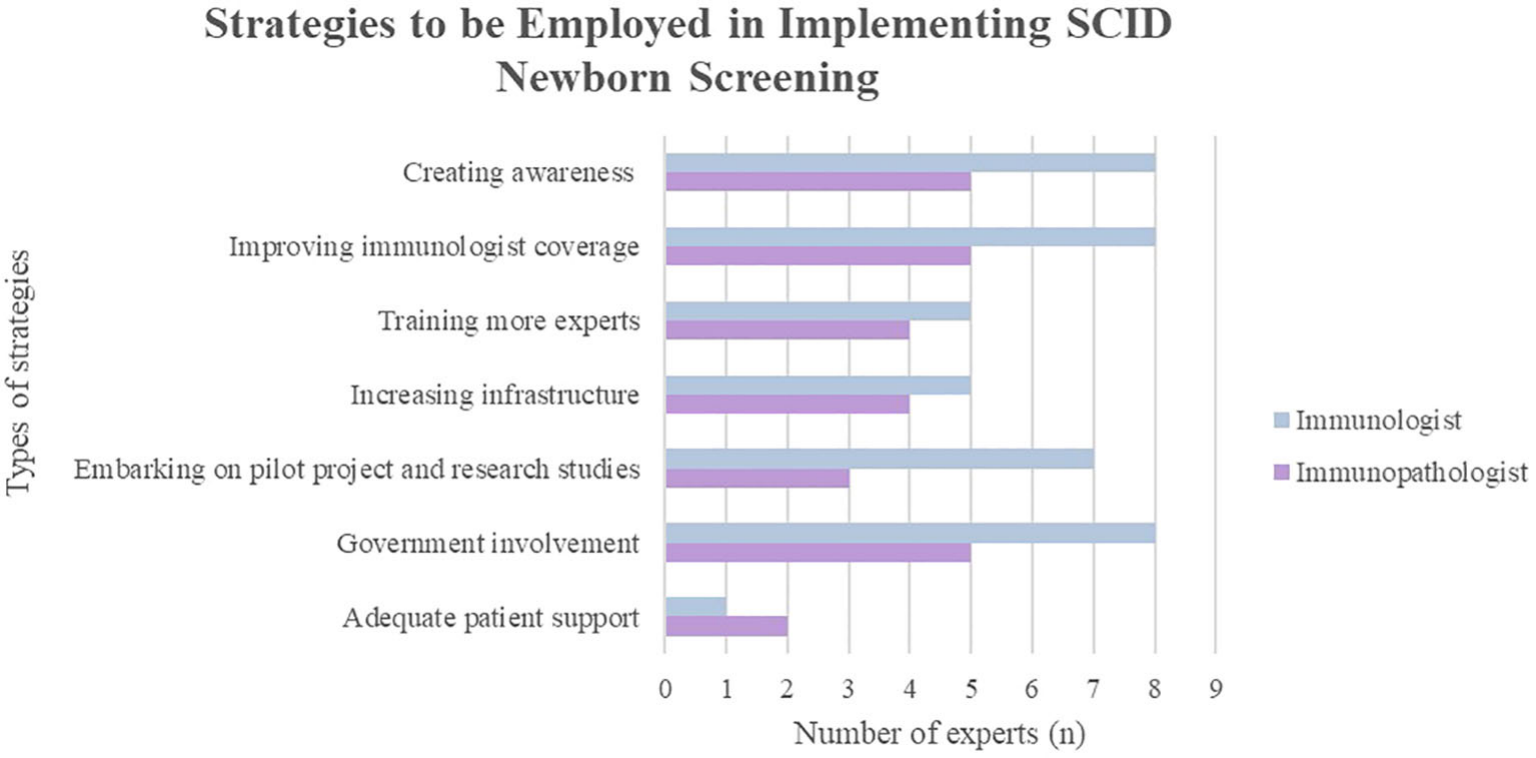
Experts’ opinion on the strategies or efforts that should be employed for successful implementation of SCID newborn screening in Malaysia.

These strategies (**Figure 2**) are only attainable through a multi-stakeholder collaboration. It is commendable that some of these efforts are ongoing, such as raising awareness, increasing PID referral centers and proposing to engage in a public-private partnership.

#### 3.3.1 Creating awareness among healthcare professionals, the public and policymakers

All the experts (100%) agree that increasing awareness among healthcare workers, policymakers, and the public is crucial. Healthcare professionals and professional institutions play a role in emphasizing the need for SCID NBS and highlighting the feasibility of its implementation through workshops, forums, and regional conferences. Academic institutions should integrate PID into the medical curriculum of undergraduate and postgraduate clinical programs to broaden their knowledge on this topic.

Educating expecting parents on SCID and the importance of NBS can be achieved through counseling, providing brochures at healthcare facilities, disseminating educational videos or online resources, and utilizing mass media platforms. To ensure routine delivery of information to expecting parents, discussions on NBS should be integrated into prenatal care protocols.

Advocacy campaigns with patron appointments and media coverage can be led by MyPOPI to raise awareness of the importance of SCID NBS and overcome the misconception that SCID is rare. Workshops and roundtable discussions involving the policymakers should be conducted to highlight the mortality rates of SCID, which are significantly contributed by delays in diagnosis or misdiagnosis.

#### 3.3.2 Ensuring adequacy of manpower and expertise

To improve the immunologist coverage in Malaysia and acknowledge the critical importance of the immunology field, initiatives should focus on increasing training opportunities for more immunologists, facilitating the involvement of visiting immunologists, and strengthening cooperation between government and university hospitals. Regional multidisciplinary groups can be formed to discuss complex cases or diagnostic dilemmas with expert input from immunologists. However, the aforementioned strategy would be stifled if the nation’s governing body for recognition of specialists, National Specialist Registry (NSR) continues to delay the recognition of clinical immunology as a subspecialty, without which trained clinical immunologists function without credence in Malaysia.

Furthermore, academic institutions should emphasize comprehensive training to nurture more skilled clinicians, pediatricians, neonatologists, nurses, genetic counselors, and laboratory personnel capable of conducting SCID NBS. Additionally, specialized training for pediatric transplant specialists who can focus on HSCT for SCID patients is essential. Manufacturers of SCID NBS testing kits can provide training for laboratory personnel to ensure precise interpretation and prompt reporting of results.

#### 3.3.3 Increasing infrastructure

To improve accessibility for SCID patients, centers of excellence with diagnostic and transplant expertise should be established evenly throughout Malaysia. Such centers would ensure that SCID patients receive timely and accurate diagnoses, appropriate treatment plans, and continuous monitoring.

In these centers, resources should be allocated for comprehensive patient care, which includes beds, isolation wards, the organization of specialized outpatient and inpatient clinical care teams, and the development of hospital protocols. Laboratory equipment and space should be adequately prepared, with potential funding from manufacturers in a public-private partnership, to meet the country’s demands in establishing SCID NBS (14).

#### 3.3.4 Embarking on pilot projects and research studies

Most experts opined that initiating a pilot project and research studies is crucial. Collaborative efforts between academic institutions and professional societies can lead to a coordinated national effort. These studies help assess cost-effectiveness and advocate for NBS implementation.

Future plans involve academia researching new methodologies for NBS of SCID and other IEI, which can be translated into more cost-efficient homegrown diagnostic kits. This approach reduces dependence on imported products, which may incur additional logistic costs.

#### 3.3.5 Government involvement

The government’s support is crucial in ensuring the successful implementation of SCID NBS (100%). With proper acknowledgment and support, collaboration between government agencies is imperative. This includes expanding federal funding, increasing the number of screening laboratories, supporting pilot studies and research in SCID, and harmonizing data integration between the MOH and university hospitals.

Leveraging existing government infrastructure becomes possible. Pilot projects for NBS can be initiated in government hospitals with close academic partnerships, and existing policies and procedures may act as a foundation, enabling the prompt adoption of a new NBS program (24).

Additionally, recognition of clinical immunology in the National Specialist Registry (NSR) under the Malaysia Medical Council will encourage more specialists to pursue this subspecialty. Policymakers should engage all stakeholders in developing standardized national screening protocols and guidelines as well as policies and legislation to mandate SCID NBS.

#### 3.3.6 Adequate patient and NGO support

Professional societies are crucial in actively engaging patients and their families by organizing programs and support services tailored to their unique needs. These may include educational workshops, resource centers, and family counseling sessions to raise awareness and empower families with the skills and knowledge to advocate for better healthcare services.

In addition, MyPOPI can be instrumental in offering emotional and financial support to affected families. This can be done through helplines, peer support groups, provision of advocacy training, volunteer programs, and financial planning workshops to alleviate the psychological burden associated with managing a chronic condition like SCID. Financial support can help cover the cost of medical treatments, travel expenses for hospital visits, and other related expenditures that might otherwise be a significant burden.

A strong demand from patients and the public can significantly influence public support and improve policy outcomes. Through their collective voice, the importance of SCID NBS can be highlighted, paving the way for enhanced public health initiatives and improved patient care.

## 4 Conclusion

Substantial SCID cases have been diagnosed in Malaysia over the recent years, prompting a reevaluation of its perceived rarity, which could be achieved by implementing NBS for SCID. This article highlights the main barriers and challenges perceived by experts managing SCID in the effort to implement NBS for SCID in Malaysia which mainly stem from a lack of awareness even within the medical fraternity and among stakeholders. This, in turn, has led to a lack of prioritization of such initiatives. The key findings from this study were also preliminary presented during the recent roundtable engagement involving multiple stakeholders in Malaysia, receiving positive feedback and a call for action being made at the meeting. The implementation of an extended NBS program which includes NBS for SCID, Inborn Errors of Metabolism and Spinal Muscular Atrophy through a public-private partnership has been proposed and is currently being evaluated. Aligning with the recent adoption of the NBS resolution during the 77th World Health Assembly in May 2024 held in Geneva, this article also gathered and outlined strategies that the experts believe are necessary to enable the implementation of NBS program for SCID in Malaysia and countries with similar background challenges.

## Data Availability

The original contributions presented in the study are included in the article/supplementary material. Further inquiries can be directed to the corresponding author.
